# Reevaluating the beautiful is moral stereotype by examining the impact of personal liking and belief in a just world

**DOI:** 10.1038/s41598-025-97022-2

**Published:** 2025-04-11

**Authors:** Konrad Bocian, Raluca Diana Szekely-Copîndean, Katarzyna Myslinska-Szarek, Bogdan Wojciszke

**Affiliations:** 1 Department of Psychology in Sopot, SWPS University, ul. Polna 16/20, Sopot, 81-745 Poland; 2 Department of Psychology, Babeş-Bolyai University, Cluj-Napoca, Romania; 3 Department of Social and Human Research, Romanian Academy, Cluj-Napoca, Romania

**Keywords:** Attractiveness, Attitudes, Liking bias, Moral character, Stereotypes, Psychology, Human behaviour

## Abstract

According to the “beautiful is moral” stereotype, people perceive attractive individuals as more moral than unattractive ones. To refine this stereotype, we examined liking as a mediator and belief in a just world (BJW) as a moderator of this effect. Study 1 (*N* = 788; US sample) and Study 2 (*N* = 1913; Polish sample) confirmed that highly attractive individuals were perceived as more moral than moderately attractive ones, but only for female targets. As predicted, liking mediated the relationship between attractiveness and moral character judgments, supporting an affect-based explanation of this stereotype. In Study 3 (*N* = 1024; British sample), we independently manipulated the target’s attractiveness and liking and found that high attractiveness alone was no longer associated with greater morality. However, confirming the role of attitudinal influences, the liked target was judged as more moral than the disliked one. Across all three studies, BJW did not moderate the relationship between attractiveness and moral judgments, suggesting that the effect of attractiveness on morality operates primarily through affective mechanisms rather than belief in fairness or justice. These findings indicate that liking exerts a stronger influence on perceived morality than attractiveness, challenging traditional conceptions of the “beautiful is moral” stereotype.

**Supplementary Information:**

The online version contains supplementary material available at 10.1038/s41598-025-97022-2.

## Introduction

Do we judge attractive people as more moral than unattractive ones? An early seminal study by Dion and colleagues^1^ showed that attractive people were perceived as having more socially desirable personality traits compared to unattractive people, a finding known as the “beauty is good” stereotype. This aligns with the broader halo effect which suggests that a person’s overall impression—often based on a single salient characteristic such as physical attractiveness—can influence judgments of unrelated traits, including morality^2^.

However, research on attractiveness-based moral judgments has yielded mixed findings, suggesting that the application of the halo effect to moral perception may be more conditional than previously assumed. Eagly et al.^3^ conducted a meta-analysis of 76 studies and found that while attractiveness strongly influenced ratings of social competence and intelligence, its effect was weak for moral integrity and non-existent for perceptions of concern for others. Similarly, in their meta-analysis of 102 studies on adults and children, Langlois et al.^4^ concluded that attractiveness’ influence on moral trait inference had never been directly tested in a systematic manner.

More recent research has begun to examine whether physical attractiveness specifically influences moral perception. While some findings suggest that attractive individuals are perceived as more moral^5^, others indicate that excessive attractiveness can trigger negative judgments, such as perceptions of vanity or dishonesty^6^. This suggests that attractiveness may not always enhance moral evaluations but could also evoke stereotype reversals, where extremely attractive individuals are seen as manipulative or self-centered rather than morally superior. Additionally, Cui et al.^7^ showed that people with attractive faces, compared to those with unattractive faces, receive higher scores on the dimension of moral beauty but not moral goodness. Finally, moral behavior and facial appearance improve the perception of facial attractiveness^8^. Thus, the relationship between attractiveness and moral judgment may not be as straightforward as classic halo effect models suggest.

Instead, emerging evidence indicates that the context, perceived personality traits, and cultural factors moderate when and how attractiveness influences social and moral perceptions^9,10^. Moreover, although evidence indicates that attractiveness biases the moral perception of others, we argue that the current knowledge about the “beautiful is moral” stereotype is deficient and needs further clarification. Our study builds on this past literature by investigating whether the “beautiful is moral” stereotype impacts moral perception consistently across different cultures and whether it is mediated by liking rather than direct inferences about character. Moreover, we aim to test belief in a just world as a moderator of the “beautiful is moral” stereotype, as past research found this belief to moderate the “beauty is good” stereotype^11^.

## Liking as a mediator of the “beautiful is moral” stereotype

Han and Laurent^6^ showed that attractiveness impacts inferences about traits related to morality via perceptions of other traits, such as sociability and vanity. Sociability has been well-established as a core component of warmth, which is one of the two fundamental dimensions of social cognition^12^. Because warmth is closely linked to perceptions of morality^13^, it is important to examine whether sociability acts as a bridge between attractiveness and moral character attributions. In contrast, vanity represents a negative trait associated with self-absorption and narcissism^14^, which could lead to a stereotype reversal effect^15^. If attractive individuals are perceived as vain, this may attenuate or even invert the “beautiful is moral” stereotype. Therefore, testing sociability and vanity allows us to determine whether the effect of attractiveness on moral judgments is purely aesthetic or whether it is mediated by inferences about social personality traits.

However, we argue that attitudes, which are complex evaluations of objects, people, groups, issues, or concepts^16^, may also explain why attractiveness impacts moral character judgments of others apart from the inference of sociability and vanity traits. Specifically, based on past research on attitudinal influences on attributions of moral character^17^, we propose that the influence of attractiveness on moral character judgments could be explained by the observer’s liking changes toward a judged person.

On the one hand, when presented with information regarding the inherent qualities or features of someone or something, people can make inferences about personality traits associated with them^18^. However, they can also infer personality traits based on physical appearance^19^ or facial expressions^20^. On the other hand, people can infer personality traits based on their attitudes toward a target person, as attitude formation is a distinct psychological process from trait inferences^21^.

Drawing on theories of attitude formation and dual-process models of moral judgment, Bocian and colleagues^17^ found that subjective factors, particularly attitudes, significantly affect moral judgments. Through various manipulations of attitudes, including the similarity or dissimilarity of beliefs, mere exposure, and facial mimicry, their findings underscore the powerful role attitudes play in shaping perceptions of moral character. Furthermore, they revealed that these effects were mediated by shifts in the perceived likability of the individuals being judged, indicating that attitudinal factors are central to how moral character is perceived^17^.

Interestingly, even when additional information about the target person’s behavior (moral or immoral) is provided, liking still significantly impact moral character perceptions more than the behavior itself^22^, even when people are asked not to use liking in their moral judgments^23^. Correspondingly, a recent study confirms the impact of liking on trait inference, as a target’s likeability was a more important predictor of the target’s authenticity than the target’s behavior or personality traits^24^.

Therefore, we propose that liking substantially impacts moral character judgments more than trait inference. We base our prediction on evidence that people automatically evaluate objects as positive or negative^25^, and these evaluations, like affective changes, can emerge and operate without conscious awareness^26^. As a result, attitude valence (positive or negative) could be a vital source of inference about others’ traits as it is processed automatically, effortlessly, and beyond conscious awareness. Research on egocentric evaluation corresponds with this premise as our unique point of view is easily and automatically accessible, contributing to errors in moral cognition^27; 28^.

Since inferences based on attitudes are generated automatically, we presumed that when people see an attractive person, an attitude is built first and trait inference second. This suggests that liking plays a critical role in shaping the pathway from attractiveness to moral character judgments. If so, attitudes could be measured as the extent to which people like the judged person, and this liking would mediate the relationship between attractiveness and moral character judgments—both directly and via inference of sociability and vanity traits.

Measuring liking, sociability, and vanity together is important for several reasons. First, sociability enhances warmth-based moral attributions through its positive impact on liking. Second, vanity may weaken or even reverse the “beautiful is moral” stereotype due to its potential to reduce liking. Third, in social psychology, numerous empirical studies have confirmed that morality is the most crucial factor in determining likability^29; 30^. Furthermore, liking and morality are so closely linked that their relationship cannot be suppressed even when manipulated independently^31^. Finally, understanding the sequential process—from attractiveness to attitudes (liking) to personality inference—allows us to refine existing models of attractiveness-based social cognition by highlighting the distinct but interrelated pathways that shape moral judgments.

In conclusion, we have substantial evidence that liking and morality are connected and that liking significantly affects the perception of moral traits and behaviors. Additionally, past research indicates that attractiveness influences not only trait inference^3^ but also liking^32;33^, suggesting that attractiveness, liking, and morality are interrelated and mutually influence one another. However, based on past research showing that both attractiveness and liking impact moral attributions, we sought to test the exclusive path in which attractiveness impacts moral character attributions via liking changes toward the judged person. In addition, we aimed to test whether the liking mechanism links attractiveness with morality via sociability and vanity.

## Belief in a just world as a moderator of the “beautiful is moral” stereotype

In a 1987 paper, Dion and Dion^11^ proposed an intriguing explanation of why attractive individuals are perceived as holding more socially desirable personality traits than unattractive individuals. Based on the just-world hypothesis^34^, they assume that attractiveness stereotyping resembles people’s belief in a just world. The just-world hypothesis proposes that humans inherently desire to believe that the world operates fairly and equitably, where individuals receive what they deserve. According to the just-world hypothesis, people tend to favor “winners,” even if they only won by chance or their only attribute is that they are physically attractive^11^.

For example, one study showed that people felt less comfortable when, at random, an unattractive worker received a reward instead of the attractive worker, although they performed tasks equally. In other words, because observers expected an attractive worker to deserve more payment than the unattractive one, their belief in a just world was disrupted, and they felt uneasy^34^. Thus, Dion and Dion^11^ proposed that those who believe in a just world are more likely to stereotype people based on their attractiveness than those who do not believe in a just world. To test this idea, they ran an experiment in which participants judged the personality of attractive and unattractive women and men targets. They also measured participants’ belief in a just world.

The study’s results confirmed that attractive targets were believed to have more socially desirable personalities (“the beauty is good” stereotype). Interestingly, the effect of attractiveness was influenced by the gender of the person being evaluated and the participant’s belief in a just world. Participants who believed in a just world perceived attractive men as having more socially desirable personalities than those who did not believe in a just world. However, no effect was found for women targets^11^. Thus, these results confirmed that “the beauty is good” stereotype held only among participants who believed that the world is a just place.

However, whether belief in a just world moderates the impact of attractiveness on moral trait perception is an open, empirical question. Answering this question is important for two reasons. First, to our knowledge, this moderation effect was not tested again after the first publication of Dion and Dion^11^. Second, although this is the highly relevant and possible moderator of the “beauty is moral” stereotype, as suggested by the current state of the art, we lack data that could confirm or disconfirm if people’s beliefs about a just world moderate the impact of attractiveness on moral trait perception. Therefore, we sought to fill this critical gap in knowledge by testing if belief in a just world moderates the “beauty is moral” stereotype.

Recent work has refined the conceptualization and measurement of belief in a just world, distinguishing between its general and personal dimensions^35;36;37^. General BJW refers to the belief that the world operates fairly—people, in general, get what they deserve. This aligns closely with the BJW measure used in Dion and Dion’s^11^ study. However, research has shown that personal BJW—the belief that one’s own life is just and that one personally receives fair treatment—is a separate construct with distinct psychological implications^35^. Individuals with strong personal BJW tend to report greater well-being, resilience, and motivation, while those with strong general BJW are more likely to endorse system-justifying beliefs and social hierarchies^38;39^.

Since these two dimensions of BJW influence social judgments differently, our study measured them separately to determine their respective roles in moderating the “beauty is moral” stereotype. Given prior research indicating that general BJW is linked to greater stereotyping and preference for socially dominant individuals^39^, we predicted that general BJW would amplify the association between attractiveness and perceived morality. However, it remains an open question whether personal BJW—which reflects self-focused just-world beliefs—plays a similar role in attractiveness-based moral judgments. By incorporating these more refined theoretical and measurement approaches, our study aimed to clarify whether belief in a just world moderates the impact of attractiveness on moral trait perception, filling a critical gap in the literature.

## Overview of present studies

We conducted three preregistered experiments with samples from the USA, Poland, and the UK to test our predictions. We presented participants with photographs of moderately or highly attractive white women and men drawn from the Face Research Lab London Set^40^. We also measured the target’s perceived sociability, vanity, and morality using scales from Study 2a by Han and Laurent^6^. Because moral traits are a perception of moral character and, as documented by different research groups, dominate impression formation^41;42;30^ we use the term *moral character* when we write about the perception of morality and moral traits. We measured participants’ liking toward moderately and highly attractive targets to test the psychological mechanism as the potential explanatory factor for how attractiveness biases moral character judgments beyond inference of sociability and vanity. To examine if the “beautiful is moral” stereotype would be moderated by levels of general and personal belief in a just world, we used the scale developed by Dalbert^35^.

## Study 1

In Study 1, we aimed to extend the current knowledge on the “beautiful is moral” stereotype by testing participants’ beliefs in a just world (moderator) and participants’ liking toward judged targets (mediator). We assumed that highly attractive targets would be considered as having more moral character than moderately attractive targets (Hypothesis 1). Moreover, we hypothesized that participants’ belief in a just world would moderate the main effect of attractiveness on moral character judgments. Hence, attractive targets would be seen as moral only in the eyes of believers in a just world (Hypothesis 2). Further, we measured participants’ attitudes by asking them to what extent they liked highly and moderately attractive targets, presuming that liking would predict moral character judgments when controlling for warmth and vanity (Hypothesis 3). Finally, we assumed liking would mediate the relationship between attractiveness manipulation and moral character judgments (Hypothesis 4). All hypotheses were preregistered at https://aspredicted.org/DRT_WGR.

## Method

This article reports all measures, all manipulations, and any data exclusions. For each study, Supplementary Materials are openly available on the Open Science Framework (osf.io/c8d4g*).* They include the preregistrations, power analysis code, and output, and for each study, verbatim instructions and stimuli, data processing and analysis code, as well as the .html output with both main (before and after outlier exclusions) and preregistered exploratory analyses results. The reported studies were approved by the ethical committee (Ethics Clearance ID: WKE/S 2022/17/X/121) and were performed by the guidelines and regulations of the Institutional Ethics Committee at the Faculty of Psychology. All participants provided informed consent.

### Participants

The Smallest Effect Size of Interest (SESOI) found in Han and Laurent’s (2022) Study 2a was *d* = 0.20. Using G*Power^41^, we estimated a total sample size of 786 participants to replicate this effect, with a given alpha of 0.05 and power of 0.80. Using Prolific Academic, we recruited 818 U.S. participants (394 women, 389 men, 20 other, 15 missing, *M*_age_ = 37.96 years, *SD* = 12.94) to participate in an online study about attractiveness. From this sample, 17 participants were excluded due to not finishing the survey (i.e., most responses were missing). We applied only one preregistered exclusion criterion (failing one attention check), which resulted in 4 exclusions. The other preregistered criterion (spending less than 5 min to fill in the survey) was not applied because it led to too many exclusions (initial *N* = 818, final *N* = 484) and did not meaningfully change the results. The final sample consisted of 788 participants (389 women, 379 men, 20 others, *M*_age_ = 38.01 years, *SD* = 12.97). In this sample, 74.8% of participants identified as White, 9.3% as Black, 7.1% as Asian, 6.1% were of mixed ethnicity, and 2.8% responded as having other ethnicities. Based on a sensitivity power analysis conducted with G*Power^43^, this sample size provides a power of 0.80 to detect a main effect size of *d* = 0.20.

### Design and procedure

We used materials from Study 2a of Han and Laurent^6^ for attractiveness manipulation. Participants were randomly assigned to one of two conditions: moderately vs. highly attractive men and women. In both conditions, participants saw one man and one woman face in random order (see Supplementary Materials). After each face was presented, we asked participants about the pictured person’s attractiveness, their attitude toward the pictured person measured as liking, and then sociability, vanity, and moral character (randomized). Finally, we measured participants’ personal and general beliefs in a just world.

### Measures

Target attractiveness was measured with the item: “To what extent would you say that this person is physically attractive?”. Participants indicated their answers using a 7-point scale from 1 = *not at all* to 7 = *very* (*M* = 4.44, S*D* = 1.45).

Sociability of the target person was measured with five items: sociable, happy, agreeable, easy-going, and playful). Participants indicated the extent to which they agreed that the target person has these five traits using a 7-point scale from 1 = *not at all* to 7 = *extremely* (*α* = 0.86, *M* = 4.02, *SD* = 0.98).

Vanity of the target person was measured with three items: vain, egotistical, and self-centred). Participants indicated the extent to which they agreed that the target person has these three traits using a 7-point scale from 1 = *not at all* to 7 = *extremely* (*α* = 0.86, *M* = 3.99, *SD* = 1.13).

Liking toward the target person was measured with: “I like this person” and “I would like to meet this person in the future.” Participants indicated to what extent they agreed with each statement using a 7-point scale from 1 = *definitely not* to 7 = d*efinitely yes* (*α* = 0.85, *M* = 3.82, *SD* = 1.24).

The target’s moral character was measured using four positive moral traits: ethical, principled, honest, and trustworthy. Participants indicated the extent to which they agreed that the target person has these traits using a 7-point scale from 1 = *not at all* to 7 = *extremely* (*α* = 0.89, *M* = 4.15, *SD* = 0.93).

Belief in a Just World (BJW) was measured with a 13-item version of the General and the Personal Belief in a Just World Scale^35^. Sample items were: “I think basically the world is a just place.”, “Overall, events in my life are just.”. Participants responded on a scale from 1 = strongly disagree to 6 = strongly agree (personal BJW: *α* = 0.93, *M* = 3.81, *SD* = 1.11; general BJW: *α* = 0.90, *M* = 3.41, *SD* = 1.20).

### Statistical analysis

Data was screened for multivariate outliers using a Minimum Covariance Determinant approach^44^. Given that across conditions (1576 cases), there were only 107 cases of multivariate outliers, we proceeded with analyses on the full dataset. However, as preregistered, we also ran all analyses on the reduced dataset after excluding outliers (see Supplementary Materials; examining the main (confirmatory) analyses after the data were screened for multivariate outliers revealed minor changes for Studies 2 and 3, but none that would change the main conclusions. However, in Study 1, liking was no longer a statistically significant mediator (initial *p* = .023 vs. *p* = .084 after excluding multivariate outliers).

To test H1 (the “beautiful is moral” stereotype), we used a mixed analysis of variance, with attractiveness manipulation 2 (high vs. moderate) as the between-subject factor and target gender 2 (men vs. women) as the within-subject factor. For pairwise comparisons, we report Bonferroni adjusted p values. The participants’ gender was added as a covariate (−1 = men; 1 = women). For this analysis, we report estimated marginal means and standard errors. H2 (BJW as moderator of the “beautiful is moral” stereotype) was tested with multiple linear regression following recommendations by Hayes (Model 1;^45^). H3 (effect of liking), controlling for vanity and sociability) was tested with mixed effects linear models, with participant ID as a random intercept (to account for repeated measures). In step 1, we regressed attractiveness, vanity and sociability onto moral character judgments, and in step 2, we added liking as a predictor. Participant gender was added as in all these models. For H4 (liking as a mediator), we used a similar mixed effects linear model approach, following recommendations by Hayes (Model 4;^45^). Additionally (not preregistered), we ran two serial mediation models (Model 6; Hayes^45^), , with liking as the first mediator and sociability and vanity, respectively, as the second mediator, to investigate if liking explains the impact of attractiveness on moral character judgments via sociability and vanity.

All analyses were run in R version 4.2.3^46^.

## Results

Zero-order correlations (across attractiveness groups and gender conditions) are displayed in Table 1. Moral character judgments were strongly and positively correlated with target attractiveness, liking, and sociability and negatively correlated with vanity. Target attractiveness was strongly and positively correlated with liking and sociability.

**Table 1:** Correlations between focal variables (Study 1).

	1	2	3	4	5	6
1. Moral character	–					
2. Target attractiveness	0.44^***^	–				
3. Liking	0.59^***^	0.65^***^	–			
4. Vanity	− 0.33^***^	0.01	− 0.16^***^	–		
5. Sociability	0.60^***^	0.53^***^	0.58^***^	− 0.16^***^	–	
6. Personal BJW	0.20^***^	0.08^**^	0.15^***^	0.004	0.15^***^	–
7. General BJW	0.20^***^	0.07^**^	0.18^***^	− 0.05^*^	0.15^***^	0.61^***^

BJW = belief in a just world. Correlation coefficients were obtained using Pearson’s method with pairwise deletion. Significance was adjusted using the false discovery rate method for multiple comparisons^47^.

^*^p < .05, ^**^p < .01, ^***^p < .001.

Table 2 shows that manipulating attractiveness impacted perceptions of the target’s attractiveness, sociability, liking, and moral character judgments, but not vanity.

**Table 2:** Main effect of attractiveness manipulation on each dependent variable (Study 1).

	Attractiveness					
	High	Moderate				
Dependent variable	*M (SE)*	*M(SE)*	*F*	*p*	*η²p*	90% CI
Target attractiveness	4.98 (0.05)	3.94 (0.05)	184.10	< 0.001	0.19	0.15, 0.23
Sociability	4.32 (0.04)	3.74 (0.04)	120.37	< 0.001	0.14	0.10, 0.17
Vanity	4.02 (0.05)	4.00 (0.05)	0.87	0.351	0.00	0.00, 0.01
Liking	4.09 (0.05)	3.59 (0.05)	49.01	< 0.001	0.06	0.04, 0.09
Moral character	4.29 (0.04)	4.01 (0.04)	26.03	< 0.001	0.03	0.02, 0.06

Means refer to estimated marginal means. Only the main effect of the attractiveness manipulation for each dependent variable is displayed. The full model in each case included both target gender as a main effect and participant gender as a covariate. Full results are reported in the Supplementary Materials.

### Moral character

**Fig. 1:**
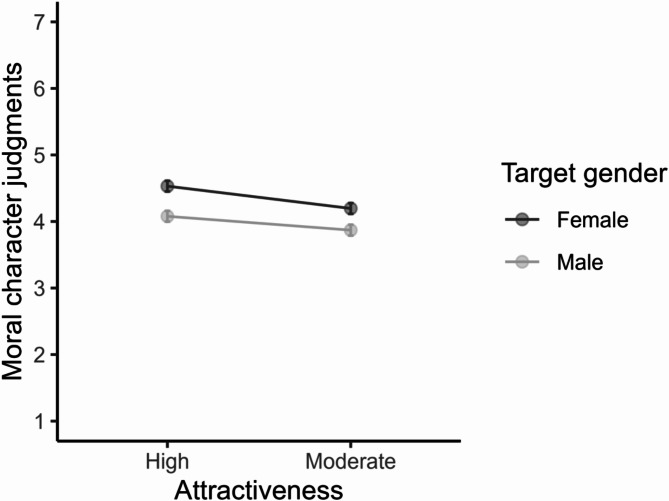
The Interaction between Attractiveness and Target Gender on Moral Character (Study 1). Note. Error bars represent 95% confidence intervals.

The “beautiful is moral” stereotype was confirmed (see Table 2), with highly attractive targets being evaluated as having higher moral character relative to moderately attractive targets, *t*(765) = 5.10, *p* < .001, *d* = 0.30, 95% CI [0.19, 0.42]. There was also a significant main effect of target gender, *F*(1, 765) = 151.14, *p* < .001, *η²*_p_ = .17, 95% CI [0.13, 0.20]. Participants rated women targets, *M* = 4.36, *SE* = 0.03, as having higher moral character than men targets, *M* = 3.95, *SE* = 0.03, *t*(765) = −12.29, *p* < .001, *d* = −0.44, 95% CI [−0.52, −0.37]. The interaction between attractiveness and target was also significant, *F*(1, 765) = 12.73, *p* < .001, *η²*_p_ = .02, 95% CI [0.01, 0.03]. Pairwise comparisons showed that the effects of attractiveness manipulation were more pronounced for women than men targets (see Fig. 1).

Highly attractive women targets, *M* = 4.56, *SE* = 0.04, were judged as significantly more moral than moderately attractive women targets, *M* = 4.16, *SE* = 0.04, *t*(765) = 6.38, *p* < .001, *d* = 0.43, 95% CI [0.25, 0.61]. No significant differences existed between highly attractive and moderately attractive men targets, *t*(765) = 2.44, *p* = .090, *d* = 0.17, 95% CI [−0.02, 0.36].

### BJW as a moderator

The effect of attractiveness on moral character judgments was not significantly moderated by either personal BJW, *F*(1, 763) = 1.72, *p* = .190, or general BJW, *F*(1, 763) = 0.19, *p* = .663.

### Liking as a predictor

Table 3 shows that liking was a significant positive predictor of moral character judgments after accounting for the effects of attractiveness, target and their interaction, sociability, vanity, and participant gender. More importantly, incorporating liking into the model eliminated attractiveness as a predictor of moral character judgment. However, adding liking as a predictor at the same time improved model fit, as both the Akaike and Bayesian information criteria were smaller for the model in Step 2 compared to Step 1 (see Table 3).

### Liking as mediator

**Fig. 2:**
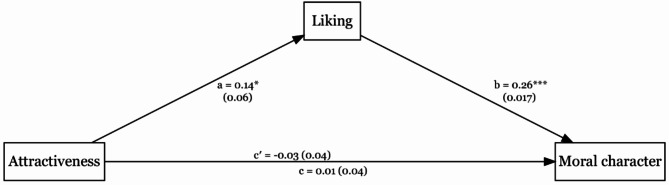
The Effect of Attractiveness on Moral Character Mediated by Liking. *Note.* Unstandardized coefficients and standard errors are displayed for each path. Path c’ represents the direct effect, while path c represents the total effect. ^*^p < .05, ^***^p < .001.

Controlling for sociability and vanity, mediation analysis showed that liking mediated the effect of attractiveness on moral character judgments, indirect = 0.04, *SE* = 0.02, *p* = .023, 95% CI _MCMC_ [0.01, 0.07]. Coefficients for each path are displayed in Fig. 2. We have tested the same model with moral character judgments as a mediator, which indicated that moral character did not mediate the effect of attractiveness on liking, indirect = 0.004, *SE* = 0.02, *p* = .849, 95% CI _MCMC_ [−0.04, 0.05].

**Fig. 3:**
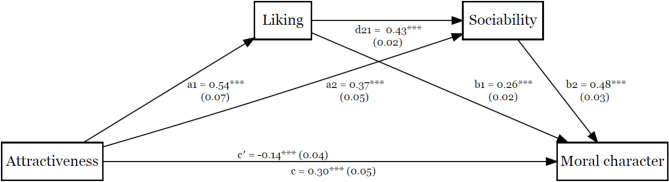
The Effect of Attractiveness on Moral Character Serially Mediated by Liking and Sociability. Note. Unstandardised coefficients and standard errors are displayed for each path. Path c’ represents the direct effect, while path c represents the total effect. ***p < .001.

**Fig. 4:**
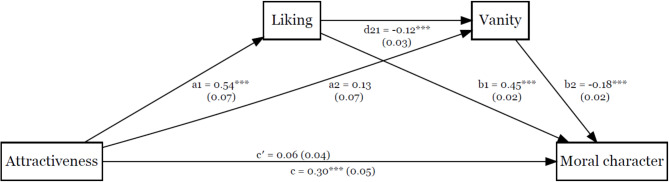
The Effect of Attractiveness on Moral Character Serially Mediated by Liking and Vanity. Note. Unstandardised coefficients and standard errors are displayed for each path. Path c’ represents the direct effect, while path c represents the total effect. ***p < .001.

The exploratory serial mediation analysis results are displayed in Figs. 3 and 4. We found the effect of attractiveness on moral character to be mediated by liking and by sociability, indirect = 0.11, 95% boot CI (0.08, 0.16). Similarly, the effect of attractiveness on moral character was mediated by liking and vanity, indirect = 0.01, 95% boot CI [0.003, 0.02].

**Table 3:** Mixed effects linear model results with predictors of moral character judgments (Study 1).

	Step 1					Step 2				
Predictors	B	SE	95% CI	Beta	*p*	B	SE	95% CI	Beta	*p*
Attractiveness (high)	0.25	0.05	0.15, 0.35	0.13	< 0.001	−0.02	0.04	−0.1, 0.06	−0.01	0.610
Target gender (men)	0.10	0.05	0.01, 0.19	0.05	0.029	−0.06	0.03	−0.12, 0.005	−0.03	0.070
Attractiveness × Target gender	−0.48	0.06	−0.59, −0.36	−0.22	< 0.001	−0.38	0.06	−0.50, −0.27	−0.18	< 0.001
Sociability	0.48	0.02	0.44, 0.52	0.51	< 0.001	0.34	0.02	0.29, 0.38	0.35	< 0.001
Vanity	−0.20	0.02	−0.23, −0.17	−0.25	< 0.001	−0.19	0.02	−0.21, −0.16	−0.23	< 0.001
Participant gender (men)	−0.01	0.02	−0.05, 0.03	−0.01	0.801	−0.03	0.02	−0.07, 0.004	−0.04	0.080
Liking						0.25	0.02	0.22, 0.28	0.33	< 0.001
	σ^2^ = 0.35					σ^2^ = 0.32				
	τ_00_ = 0.13					τ_00_ = 0.10				
	AIC = 3224.16					AIC = 3033.42				
	BIC = 3272.20					BIC = 3086.80				
	ICC = 0.28					ICC = 0.25				
	*N* = 768					*N* = 768				
	Observations = 1536					Observations = 1536				
	R^2^_marginal_ = 0.41/ R^2^_conditional_= 0.57					R^2^_marginal_ = 0.49/ R^2^_conditional_= 0.62				

The effects of attractiveness and target gender are simple effects (not main effects).

σ^2^ = residual variance, τ_00_ = variance of random intercept, AIC = Akaike information criterion, BIC = Bayesian information criterion, ICC = intraclass correlation coefficient, R^2^_marginal_ = fixed effects, R^2^_conditional_ = fixed and random effects.

## Discussion

Study 1 confirmed three of our four hypotheses. First, we replicated the “beautiful is moral” stereotype, as highly attractive targets were judged as having more moral character than moderately attractive targets. However, this effect was limited to women targets. Further, we did not find evidence that highly attractive targets were perceived as more vain than moderately attractive ones. These results contrast with previous research by Han & Laurent^6^, who did not find interactions between attractiveness manipulation and participants’ gender but found that attractiveness impacts the perception of vanity.

Second, liking predicted moral character judgments when controlling for sociability and vanity, improving model fit and explaining the additional variance of moral character judgments. Interestingly, once liking was added to the model, attractiveness was no longer a predictor of moral character judgments. Finally, liking alone mediated between attractiveness manipulation and moral character judgments, but also when sociability and vanity were added as second mediators. This evidence supports our assumption that liking is essential in how attractiveness shapes moral character, even if we account for perceptions of sociability and vanity.

Surprisingly, the personal and general BJW did not moderate the “beautiful is moral” stereotype. The lack of the moderation effect could be due to the sample being too small to detect the moderation effect and/or the kind of sample we recruited. Dion and Dion’s^11^ study, as well as Study 1, was conducted in the USA within a society with a relatively high belief in a just world. For instance, one study showed that American participants from Amazon Mechanical Turk reported mean general belief in a just world (GBJW) at the level of 3.77 while personal (PBJW) at the level of 4.26 on the 6-point scale^37^.

In contrast, data from Poland, a post-communist country, shows that Poles report GBJW at 3.10 and PBJW at 3.77^48^. This corresponds with data showing that Poles tend to disbelieve in a just world^49^. Therefore, we sought to replicate the effects found in Study 1 using a Polish sample to investigate if cultural differences in belief in a just world may impact the “beautiful is moral” stereotype.

## Study 2

Study 2 was a direct replication of Study 1 with one exception. We used a much larger sample of Polish participants to have enough power to detect even small moderation effects and to test if societally lower levels of BJW may count in the perception of attractiveness and morality. The remaining sections of Study 2 were identical to Study 1. Hypotheses were preregistered at https://aspredicted.org/CGK_TW7.

## Method

### Participants

The sample size estimation was based on the effect size we obtained in Study 1, which consisted of the partial eta squared of the interaction between the attractiveness manipulation (high, moderate) and target gender (men, women), *η*^*2*^_*p*_ = 0.016. We estimated a total sample size of 485 participants (raw estimate = 484.634). Following recommendations proposed by Giner-Sorolla^50^ for testing moderators, we estimated that our sample size should be four times larger, which resulted in *N* = 1940.

We recruited *N* = 2010 Polish participants from the Polish research panel Ariadna. From this sample, 3 participants were excluded because they reported being less than 18 years old. We applied only one preregistered exclusion criterion (failing one attention check), which resulted in an additional 94 exclusions. The other preregistered criterion (spending less than 5 min to fill in the survey) was not applied because it led to too many exclusions (initial *N* = 2010, final *N* = 1585) and did not meaningfully change the results. After exclusions, the final sample consisted of *N* = 1913 participants (850 women, 804 men, 3 other, *M*_age_ = 46.67 years, *SD* = 14.54). Based on a sensitivity power analysis conducted with G*Power, this sample size provides a power of 0.80 to detect an interaction effect size of *η*^*2*^_*p*_ = 0.002.

### Design and procedure

The research procedure in Study 2 was identical to Study 1, with the difference that all questions and descriptions presented to the participants were in Polish. Moreover, participants’ belief in a just world was randomly measured before or after manipulating and measuring dependent variables. In this way, we aimed to control if the measure of belief in a just world could influence the impact of attractiveness on moral character judgments. To measure the BJW, we used the Polish adaptation of the scale by Larionov and Mudło-Głagolska^48^.

### Measures

Target attractiveness was measured as in Study 1 (*M* = 4.07, *SD* = 1.54).

Sociability of the target person was measured as in Study 1 (α = 0.88, *M* = 3.77, *SD* = 1.08).

Vanity of the target person was measured as in Study 1 (α = 0.76, *M* = 3.74, *SD* = 1.10).

Attitude (liking) toward the target was measured as in Study 1 (α = 0.70, *M* = 3.56, *SD* = 1.30).

Moral character of the target was measured as in Study 1 (α = 0.91, *M* = 3.87, *SD* = 1.08).

Belief in a Just World (BJW) was measured as in Study 1 (personal BJW: α = 0.90, *M* = 3.64, *SD* = 1.00; general BJW: α = 0.87, *M* = 3.28, *SD* = 1.03).

### Statistical analysis

We followed a similar analysis pipeline as in Study 1. Additionally, given that approximately 18.37% of the data points in our dataset (after applying the preregistered filters) were missing, we ran a Little’s missing completely at random (MCAR) chi-squared test, which was not significant (data was missing completely at random), *χ*^2^(38367) = 20005.91, *p* = 1. For each participant and variable of interest, aggregate scores were computed only if at least half of the items were available (e.g., if more than two moral character items out of four were missing, scores were not computed). We also ran a Welch’s t-test to check whether BJW scores differed depending on whether participants completed the questionnaire before or after the main task. As preregistered, we ran the main analyses after excluding multivariate outliers from the dataset and reported results in the Supplementary Materials.

## Results

Zero-order correlations (across attractiveness groups and gender conditions) are displayed in Table 4.

**Table 4:** Correlations between focal variables (Study 2).

	1	2	3	4	5	6
1. Moral character	–					
2. Target attractiveness	0.59^***^	–				
3. Liking	0.65^***^	0.66^***^	–			
4. Vanity	− 0.12^***^	− 0.03	− 0.06^**^	–		
5. Sociability	0.76^***^	0.59^***^	0.63^***^	− 0.07^**^	–	
6. Personal BJW	0.25^***^	0.19^***^	0.19^***^	0.02	0.23^***^	–
7. General BJW	0.21^***^	0.16^***^	0.17^***^	− 0.03	0.19^***^	0.67^***^

BJW = belief in a just world. Correlation coefficients were obtained using Pearson’s method, with pairwise deletion. Significance was adjusted using the false discovery rate method for multiple comparisons (Benjamini & Hochberg, 1995).

*p < .05, **p < .01, ***p < .001.

Table 5 shows that the attractiveness manipulation impacted perceptions of the target’s attractiveness, sociability, liking and moral character judgments, but not vanity, replicating the results of Study 1.

**Table 5:** Main effect of attractiveness manipulation on each dependent variable (Study 2).

	Attractiveness					
	High	Moderate				
Dependent variable	*M (SE)*	*M(SE)*	*F*	*p*	*η²*_*p*_	90% CI
Target attractiveness	4.46 (0.05)	3.68 (0.04)	168.24	< 0.001	0.09	0.07, 0.12
Sociability	4.07 (0.05)	3.59 (0.03)	64.69	< 0.001	0.06	0.04, 0.09
Vanity	3.71 (0.07)	3.75 (0.04)	0.37	0.544	0.00	0.00, 0.01
Liking	3.78 (0.04)	3.35 (0.03)	65.38	< 0.001	0.04	0.02, 0.05
Moral character	4.08 (0.06)	3.74 (0.04)	25.01	< 0.001	0.03	0.01, 0.05

Means refer to estimated marginal means. Only the main effect of the attractiveness manipulation for each dependent variable is displayed. The full model in each case included target gender as a main effect and participant gender as a covariate. Full results are reported in the Supplementary Materials.

### Moral character

**Fig. 5:**
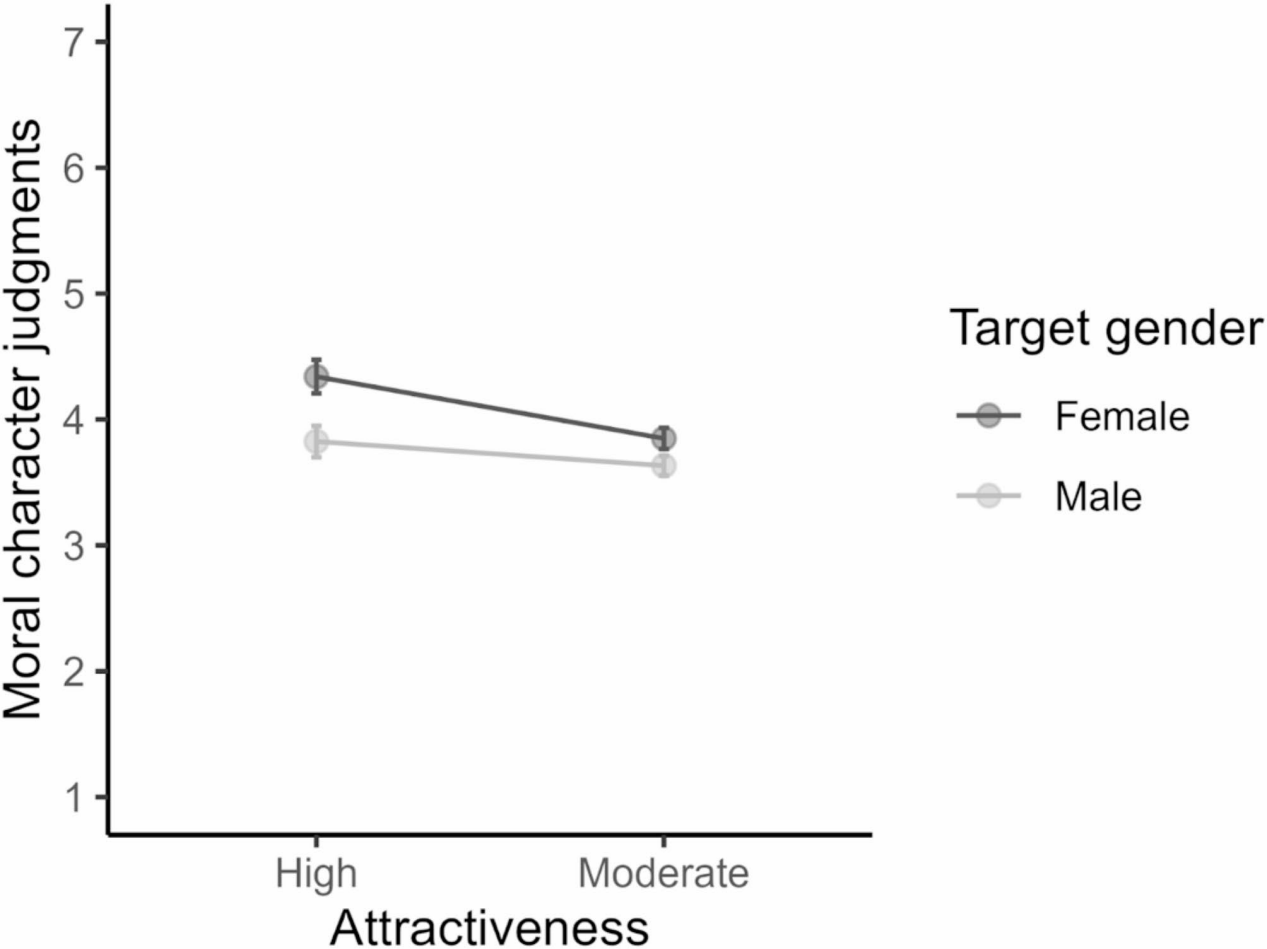
The Interaction between Attractiveness and Target Gender on Moral Character (Study 2). Note. Error bars represent 95% confidence intervals.

As in Study 1, the ”beautiful is moral” stereotype was again confirmed (see Table 5); highly attractive targets were evaluated as having higher moral character relative to moderately attractive targets, *t*(897) = 5.00, *p* < .001, *d* = 0.34, 95% CI [0.21, 0.47]. There was also a significant main effect of target gender, *F*(1, 897) = 96.03, *p* < .001, *η²*_*p*_ = .10, 90% CI [0.07, 0.13]. Participants rated men targets, *M* = 3.73, *SE* = 0.04, as having lower moral character than women targets, *M* = 4.10, *SE* = 0.04, *t*(897) = −9.80, *p* < .001, *d* = −0.36, 95% CI [−0.44, −0.29]. The interaction between attractiveness and target was also significant, *F*(1, 897) = 16.08, *p* < .001, *η²*_*p*_ = .02, 90% CI [0.01, 0.03]. Pairwise comparisons showed that attractiveness manipulation succeeded when women targets, but not men targets, were presented (see Fig. 5). Highly attractive women, *M* = 4.34, *SE* = 0.07, were evaluated as significantly more moral than moderately attractive women, *M* = 3.85, *SE* = 0.04, *t*(897) = 6.10, *p* < .001, *d* = 0.49, 95% CI [0.28, 0.70]. There were no significant differences between highly attractive men targets and moderately attractive men targets, *t*(897) = 2.56, *p* = .065, *d* = 0.19, 95% CI [−0.01, 0.39].

### BJW as a moderator

Administering the BJW scale before or after the main task was not associated with significantly different scores for either personal BJW, *t*_Welch_ (956.01) = 1.73, *p* = .080, or general BJW, *t*_Welch_ (977.33) = 1.15, *p* = .250. The effect of attractiveness on moral character judgments was not significantly moderated by either personal BJW, *F*(1, 1555) = 1.11, *p* = .292, or general BJW, *F*(1, 1548) = 0.04, *p* = .836.

### Liking as a predictor

Table 6 shows that liking was a significant positive predictor of moral character judgments, even after accounting for the effects of attractiveness, the target’s gender, and their interaction, as well as sociability, vanity, and participant gender. Again, adding liking to the model eliminated attractiveness as a predictor of moral character and improved model fit, as AIC and BIC were smaller for the model in Step 2 compared to Step 1 (see Table 6).

**Table 6:** Mixed effects linear model results with predictors of moral character judgments.

	Step 1					Step 2				
Predictors	B	SE	95% CI	Beta	*P*	B	SE	95% CI	Beta	*p*
Attractiveness (high)	0.11	0.05	0.01, 0.21	0.05	0.035	0.05	0.05	−0.05 0.14	0.02	0.314
Target gender (men)	0.05	0.04	−0.02, 0.12	0.02	0.131	0.09	0.03	0.02, 0.15	0.04	0.008
Attractiveness × Target gender	−0.28	0.06	−0.40, −0.15	−0.09	< 0.001	−0.20	0.06	−0.32, −0.08	−0.06	0.001
Sociability	0.74	0.02	0.71, 0.77	0.74	< 0.001	0.55	0.02	0.51, 0.58	0.55	< 0.001
Vanity	−0.09	0.01	−0.12, −0.06	−0.09	< 0.001	−0.08	0.01	−0.11, −0.06	−0.09	< 0.001
Participant gender (men)	0.00	0.02	−0.03, 0.03	0.00	0.988	0.002	0.02	−0.03, 0.03	0.002	0.902
Liking						0.26	0.02	0.23, 0.29	0.32	< 0.001
	σ^2^ = 0.32					σ^2^ = 0.29				
	τ_00_ = 0.15					τ_00_ = 0.11				
	AIC = 4090.48					AIC = 3812.82				
	BIC = 4140.76					BIC = 3868.68				
	ICC = 0.32					ICC = 0.28				
	*N* = 1420					*N* = 1420				
	Observations = 1972					Observations = 1972				
	R^2^_marginal_ = 0.58/ R^2^_conditional_= 0.71					R^2^_marginal_ = 0.64/ R^2^_conditional_= 0.74				

The effects of attractiveness and target gender are simple effects (not main effects).

σ^2^ = residual variance, τ_00_ = variance of random intercept, AIC = Akaike information criterion, BIC = Bayesian information criterion, ICC = intraclass correlation coefficient, R^2^_marginal_ = fixed effects, R^2^_conditional_ = fixed and random effects.

### Liking as a mediator

**Fig. 6:**
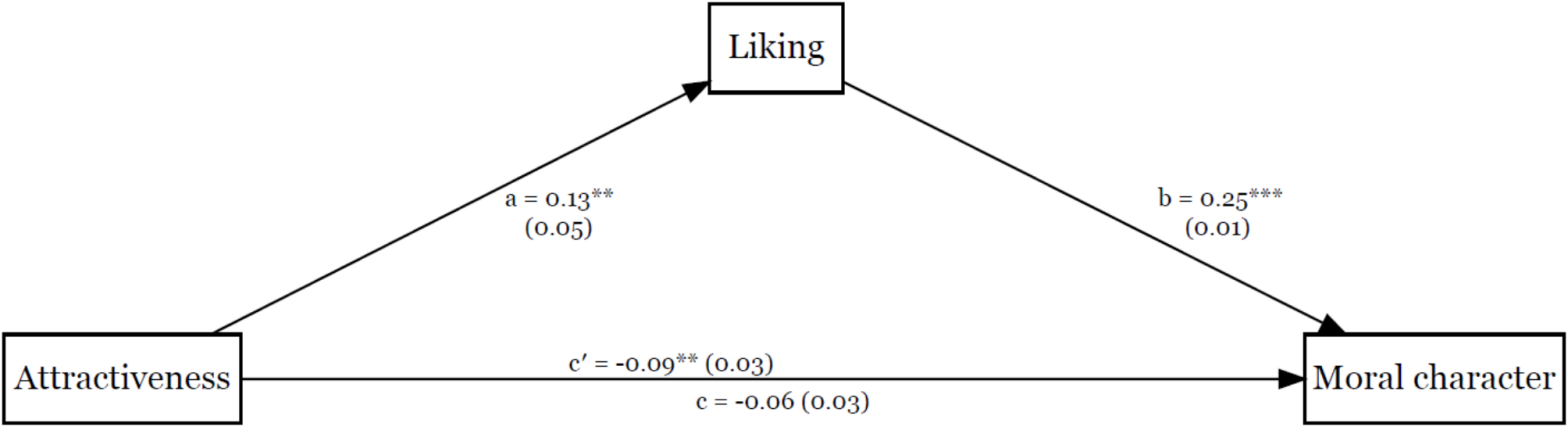
The Effect of Attractiveness on Moral Character Mediated by Liking. Note. Unstandardised coefficients and standard errors are displayed for each path. Path c’ represents the direct effect, while path c represents the total effect. ^**^p < .01, ^***^p < .001.

Controlling for sociability and vanity, we found that liking mediated the effect of attractiveness on moral character judgments, indirect = 0.03, *SE* = 0.01, *p* = .009, 95% CI _MCMC_ [0.01, 0.06]. Coefficients for each path are displayed in Fig. 6. We have tested the same model with moral character judgments as a mediator, which indicated that moral character did not mediate the effect of attractiveness on liking, indirect = 0.03, *SE* = 0.02, *p* = .115, 95% CI _MCMC_ [−0.07, 0.01].

**Fig. 7:**
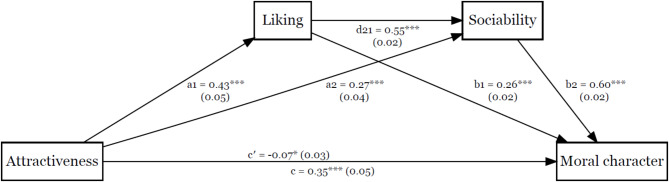
The Effect of Attractiveness on Moral Character Mediated by Liking and Sociability. Note. Unstandardised coefficients and standard errors are displayed for each path. Path c’ represents the direct effect, while path c represents the total effect. ^*^p < .05, ^***^p < .001.

**Fig. 8:**
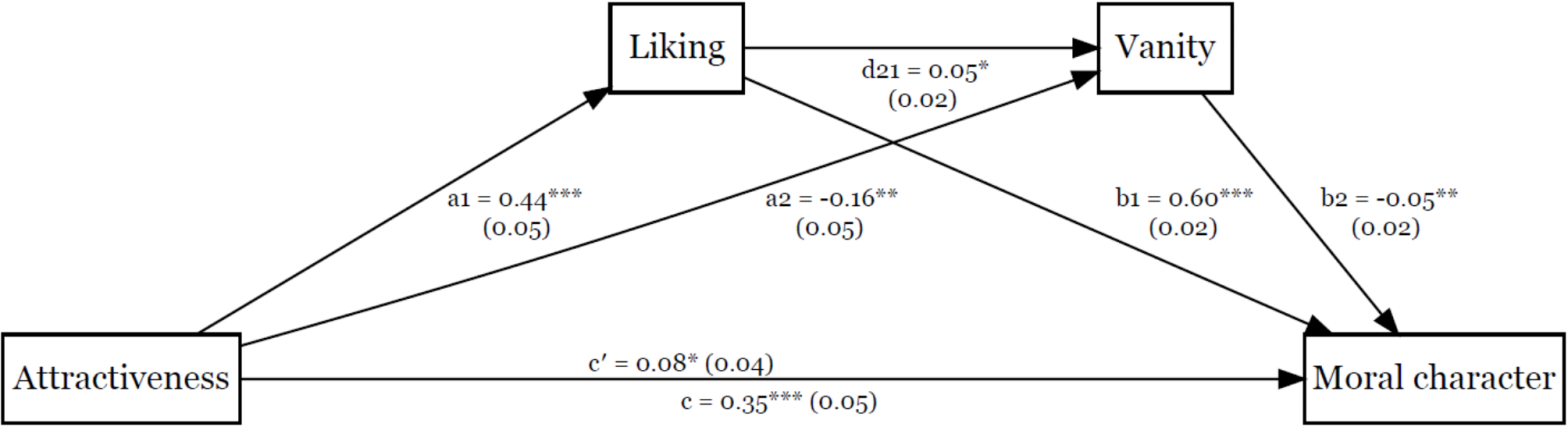
The Effect of Attractiveness on Moral Character Mediated by Liking and Vanity. Note. Unstandardized coefficients and standard errors are displayed for each path. Path c’ represents the direct effect, while path c represents the total effect. ^*^p < .05, ^**^p < .01, ^***^p < .001.

Serial mediation analysis results are displayed in Figs. 7 and 8. We found that the effect of attractiveness on moral character was mediated through liking and sociability, indirect = 0.14, 95% bootCI [0.11, 0.18]. However, the effect of attractiveness on moral character was not mediated through liking and vanity, indirect = −0.001, 95% bootCI [−0.003, 0.00].

## Discussion

Study 2 replicated some key results found in Study 1 on a culturally different sample. We again found the “beautiful is moral” stereotype, and this effect was limited to women targets. Additionally, we used an almost three times larger sample from a post-communist country where participants presented lower levels of belief in a just world than the capitalist sample. The BJW scores were higher in the US sample (personal *M* = 3.81, *SD* = 1.11; general BJW: *M* = 3.41, *SD* = 1.20) than in the Polish sample (personal BJW: *M* = 3.64, *SD* = 1.00; general BJW, *M* = 3.28, *SD* = 1.03). For the second time, we found no evidence that highly attractive targets are perceived as more vain than moderately attractive ones.

Confirming our predictions, liking predicted moral character judgments when controlling for sociability and vanity and explained additional variance of moral character judgments. Confirming the results of Study 1, when we considered liking as a predictor together with attractiveness, only liking was a significant predictor. This might suggest that liking has a stronger impact on moral character judgments and, thus, a stronger predictive effect on overriding the impact of attractiveness. Mediation models confirmed that liking alone mediated between attractiveness manipulation and moral character judgments, but also when sociability and vanity were added as second mediators. Finally, neither the personal nor general BJW moderated the “beautiful is moral” stereotype or order of the BJW scale presentation. Therefore, based on similar results from Study 1, we can conclude that there is a high probability that believing or not in a just world has no impact on the “beautiful is moral” stereotype.

To this point, we have confirmed that the “beautiful is moral” stereotype exists in the capitalistic and post-communistic cultures, but only when judging women targets. More importantly, we empirically documented that liking is another crucial factor that may help us understand how attractiveness impacts moral character judgments. However, in Studies 1 and 2, we have gathered only correlational evidence for the link between attractiveness, liking, and moral character judgments. Therefore, Study 3 tested a causal chain between attractiveness, liking, and moral character judgments by independently manipulating the likability and attractiveness of the target.

## Study 3

Studies 1 and 2 showed that attractiveness impacts moral character judgments. We propose that the psychological process that may explain this relation depends on the perceiver’s liking toward the perceived person. Specifically, we argue that liking, which strongly predict and bias moral character judgments^17^, drive the “beautiful is moral” stereotype. In Study 3, we sought to replicate earlier findings by changing the attractiveness manipulation and presenting more robust experimental evidence—beyond mere mediation—supporting liking as the basis for the “beautiful is moral” stereotype. Utilizing a “moderation-of-process” design^51^, we manipulated liking to demonstrate that the “obeautiful is moral” stereotype is constrained by attitude manipulation. We also changed the sample from Polish to British.

Extensive research indicates that similarity is the strongest factor in interpersonal attraction^52^. A meta-analysis involving over 300 lab studies revealed an average similarity-attraction correlation of *r* = .59^53^. Based on this, we manipulated the similarity between participants and a target person to manipulate participants’ liking. We assumed that similar persons would be liked more than dissimilar ones. Moreover, we theorized that the “beautiful is moral” stereotype would be moderated by similarity; highly attractive targets would be perceived as more moral when they appear similar to participants and less moral when perceived as dissimilar.

We also anticipate replicating the “beautiful is moral” stereotype and the previously observed attitudinal influences on moral character - liked people will be judged to have greater moral character than those who are not^17^. Lastly, consistent with the findings from Studies 1 and 2, we expect that participants’ belief in a just world will not affect the “beautiful is moral” stereotype. Our hypotheses have been preregistered at https://aspredicted.org/RQS_4LW.

## Method

### Participants

The sample size estimation was based on the simple effect size of the attitude manipulation impact on moral character judgments found in Study 3 by Bocian et al.^23^, *d* = 0.38 (*f* = 0.19), and the main effect size of attractiveness manipulation from our previous study, *f* = 0.17. We estimated that with a power of 0.80 and alpha of 0.05 for the interaction effect, we would need a total sample of *N* = 256. Because we assumed that attitude manipulation would knock out attractiveness manipulation, we followed the recommendations proposed by Giner-Sorolla^50^. We estimated that our sample size should be four times larger, which results in *N* = 1024.

Using Prolific Academic, we recruited *N* = 1099 British participants. From this sample, 5 participants were excluded after applying one of the two preregistered exclusion criteria (failing one attention check). The other preregistered criterion (spending less than 5 min to fill in the survey) was not applied because it led to too many exclusions (initial *N* = 1099, final *N* = 837) and did not meaningfully change the results. The final sample consisted of *N* = 1094 participants (537 women, 550 men, 7 others, *M*_age_ = 41.45 years, *SD* = 14.35). Based on a sensitivity power analysis conducted with G*Power, this sample size provides a power of 0.80 to detect a main effect size of *f* = 0.09.

### Design and procedure

The design of Study 3 was similar to Studies 1 and 2, with two exceptions. First, we changed the stimuli for the attractiveness manipulation. Specifically, we used materials from Han and Laurent^6^ from Study 2b instead of Study 2a. We used only one women target since, in Studies 1 and 2, the “beautiful is moral” stereotype was limited to women targets. Second, we added the attitude manipulation proposed by Sprecher^54^ to induce a similarity/dissimilarity between participants and the judged person. This manipulation was successfully used in the past, where the similarity with the target resulted in higher liking while dissimilarity resulted in lower liking^22^.

At the beginning of the experiment, we told participants that they would see a photo and answers from a random participant who also completed the study. Thus, we requested participants to fill out a preference questionnaire. This form consisted of 17 questions about their preferences, such as whether they preferred reality shows or sitcoms and whether they considered themselves to be dreamers or doers^54^; see Supplementary Materials for the complete list of questions). This task aimed to manipulate participants’ attitudes by convincing them that the target person is either similar or dissimilar to them, which should result in more or less liking.

Then, we told participants that a unique algorithm would now randomly draw a picture and answer from a random person who uploaded this information in the first part of the current study. We showed participants a progress bar for 14 s, suggesting the algorithm’s work. Afterward, according to the 2 (preferences: similar vs. dissimilar) x 2 (attractiveness: moderate vs. high), they saw either moderately or highly attractive women’s faces, and on the next page, their answers were similar or dissimilar to theirs. In the similar preferences condition, 14 answers from 17 mirrored participants’ responses. In the dissimilar preferences condition, 14 out of 17 answers were opposite to the participants’ responses. After participants read the answers, we asked them questions about the target used in Studies 1 and 2.

### Measures

Target attractiveness was measured as in Studies 1 and 2 (*M* = 5.51, *SD* = 1.20).

Attitude (liking) toward the target was measured as in Studies 1 and 2 (α = 0.80, *M* = 5.71, *SD* = 1.18).

Sociability of the target person was measured as in Studies 1 and 2 (α = 0.78, *M* = 4.60, *SD* = 0.85).

Vanity of the target person was measured as in Studies 1 and 2 (α = 0.83, *M* = 3.48, SD = 1.08).

Moral character of the target was measured as in Studies 1 and 2 (α = 0.85, *M* = 4.84, *SD* = 0.83).

Belief in a Just World (BJW) was measured as in Studies 1 and 2 (personal BJW: α = 0.91, M = 3.82, *SD* = 0.95; general BJW: α = 0.88, *M* = 3.21, *SD* = 1.01).

### Statistical analysis

According to the preregistered analysis plan, we followed a similar analysis pipeline as in Studies 1 and 2. All analyses were adapted for the between-subjects design (i.e., between-subjects analyses of variance and linear regression models).

## Results

Zero-order correlations (across attractiveness and attitude groups) are displayed in Table 7.

**Table 7:** Correlations between focal variables (Study 3).

	1	2	3	4	5	6
1. Moral character	–					
2. Target attractiveness	0.22^***^	–				
3. Liking	0.46^***^	0.39^***^	–			
4. Vanity	− 0.41^***^	0.05	− 0.24^***^	–		
5. Sociability	0.38^***^	0.37^***^	0.34^***^	− 0.16^***^	–	
6. Personal BJW	0.24^***^	0.08^*^	0.12^***^	− 0.08^*^	0.15^***^	–
7. General BJW	0.22^***^	0.07^*^	0.19^***^	− 0.07^*^	0.14^***^	0.62^***^

BJW = belief in a just world. Correlation coefficients were obtained using Pearson’s method, with pairwise deletion. Significance was adjusted using the false discovery rate method for multiple comparisons (Benjamini & Hochberg, 1995).

*p < .05, **p < .01, ***p < .001.

For each dependent variable, Table 8 shows the main effects of attractiveness and attitude on each dependent variable. There were no significant interaction effects (see Supplementary Materials for full results).

**Table 8:** Main effects of attractiveness and similarity manipulations on each dependent variable (Study 3).

Dependent variable	Attractiveness					Preferences				
	High	Moderate				Similar	Dissimilar			
	M (SE)	M(SE)	F	*p*	η²_*p*_ (90% CI)	M (SE)	M(SE)	F	*p*	η²_*p*_ (90% CI)
Target attractiveness	5.96 (0.05)	5.07 (0.05)	169.59	< 0.001	0.14 (0.11, 0.17)	5.59 (0.05)	5.44 (0.05)	4.94	0.026	0.01 (0.00, 0.01)
Liking	5.75 (0.05)	5.68 (0.05)	1.10	0.294	0.001 (0.00, 0.01)	6.10 (0.05)	5.32 (0.05)	132.678	< 0.001	0.11 (0.08, 0.14)
Sociability	4.64 (0.04)	4.57 (0.04)	2.06	0.151	0.002 (0.002,0.02)	4.52 (0.04)	4.68 (0.04)	10.51	0.001	0.01 (0.002, 0.02)
Vanity	3.80 (0.04)	3.15 (0.04)	111.69	< 0.001	0.09 (0.07, 0.12)	3.32 (0.04)	3.63 (0.04)	26.35	< 0.001	0.02 (0.01, 0.04)
Moral character	4.73 (0.04)	4.96 (0.03)	21.68	< 0.001	0.02 (0.01, 0.04)	5.00 (0.04)	4.69 (0.04)	40.06	< 0.001	0.04 (0.03, 0.06)

Means refer to estimated marginal means. Only the main effects of the attractiveness and attitude manipulations for each dependent variable are displayed. No interactions were significant. The full model in each case included participant gender as covariate. Full results are reported in the Supplementary Materials.

### Attractiveness and liking

Target attractiveness was rated significantly higher in the highly attractive condition relative to the moderately attractive condition, *t*(1082) = 13.02, *p* < .001, *d* = 0.79, 95% CI [0.67, 0.91]. Target attractiveness was also rated significantly higher in the similar relative to the dissimilar condition, *t*(1082) = 2.22, *p* = .026, *d* = 0.14, 95% CI [0.16, 0.25]. Conversely, liking ratings were significantly higher in the similar relative to the dissimilar attitude condition, *t*(1082) = 11.52, *p* < .001, *d* = 0.70, 95% CI [0.58, 0.81], but were not significantly different between the high and moderate attractiveness conditions, *t*(1082) = 1.05, *p* = .294, *d* = 0.06, 95% CI [−0.06, 0.18].

Table 8 also shows that sociability ratings were not significantly different depending on the level of attractiveness, *t*(1082) = 1.44, *p* = .151, *d* = 0.09, 95% CI [−0.03, 0.21], and were slightly higher in the dissimilar attitude condition relative to the similar attitude condition, *t*(1082) = −0.17, *p* = .001, *d* = −0.20, 95% CI [−0.32, −0.08]. However, vanity ratings were significantly higher for highly attractive targets, relative to moderately attractive ones, *t*(1082) = 10.57, *p* < .001, *d* = 0.64, 95% CI [0.52, 0.76], and significantly higher for targets with dissimilar attitudes, relative to similar ones, *t*(1082) = −5.13, *p* < .001, *d* = −0.31, 95% CI [−0.43, −0.19] (see Table 7).

### Moral character

Unlike in Studies 1 and 2, the “beautiful is moral” effect was not confirmed, as moderately attractive targets were judged as having higher moral character relative to highly attractive targets, *t* (1082) = −0.23, *p* < .001, *d* = −0.28, 95% CI [−0.40, −0.16]. There was also a significant main effect of preferences. Participants in the similar preferences condition relative to those in the dissimilar preferences condition judged targets as having higher moral character, *t* (1082) = 6.33, *p* < .001, *d* = 0.38, 95% CI [0.27, 0.50], see Table 8.

### BJW as a moderator

The effect of attractiveness on moral character judgments was not significantly moderated by either personal BJW, *F*(1, 1082) = 0.23, *p* = .633, or general BJW, *F*(1, 1082) = 0.01, *p* = .913.

## Discussion

Disconfirming our preregistered hypothesis and findings from Studies 1 and 2, Study 3 did not replicate the “beautiful is moral” stereotype. Although we found the effect of attractiveness manipulation, the direction of this effect was reversed, meaning that the moderately attractive target was judged as having higher moral character relative to the highly attractive target. We also did not find a predicted interaction effect for attractiveness and attitude manipulations. None of the measured dependent variables yielded interaction effects. Instead, we found two main effects, the first related to attractiveness and the second to preferences.

For attractiveness manipulation, in contrast to Studies 1 and 2, we did not find the highly attractive target to be more likable or sociable than the moderately attractive one. However, we found that the former was vainer than the latter, which corresponds with the findings of Han and Laurent^6^. For liking manipulation, we found that similar targets were perceived as more attractive, likable, and moral but less vain and sociable than dissimilar targets. Finally, we did confirm that neither the personal nor general BJW moderated the “beautiful is moral” stereotype.

One could argue that changed stimuli could reverse the “beautiful is moral” stereotype in Study 3. However, this explanation has a slight chance of being true. First, the stimulus in Study 3 was used successfully in previous studies by Han and Laurent^6^, where the target with an attractive face was judged as more moral than the target with a moderately attractive face. Second, we used the Face Research Lab London Set^40^, for which all images were taken in London, UK. Finally, as can be read in Table 8, although attractiveness manipulation was successful, it impacted morality in the opposite direction. Thus, if the attractive face was judged more attractive than a moderately attractive one, why did it not produce the “beautiful is moral” stereotype?

We argue that because the attractiveness manipulation was paired with liking manipulation (liking vs. disliking), which suppressed the impact of attractiveness (high vs. moderate) on moral trait perception. Evidence from the current three studies supports this conclusion. First, in each study, the correlation between liking and morality was stronger than the correlation between liking and attractiveness. Second, in Studies 1 and 2, attractiveness stopped predicting morality when attitudes liking was added to the model. Therefore, it seems correct to assume liking have a stronger impact on moral character attributions than attractiveness.

## General discussion

In this research, we aimed to contribute to the limited literature on how attractiveness affects moral character attributions. We found the “beautiful is moral” stereotype in American and Polish samples but not in a British sample. However, the effect was limited to women targets. We also found evidence that liking predicts moral character judgments when controlling for sociability and vanity. Finally, in Studies 1 and 2, liking mediated the impact of attractiveness on moral character judgments alone and when sociability and vanity were considered second mediators. In Study 3, we manipulated whether the target’s preferences were either similar or dissimilar to those of the participants and showed that independent from attractiveness, the target with similar preferences was liked more and perceived as more moral than the target with dissimilar preferences. Interestingly, in Study 3, we did not find evidence for the interaction between attractiveness and liking manipulation. However, we discovered that the “beautiful is moral” stereotype was reversed, so moderate attractiveness was more moral than high attractiveness when similar or dissimilar attitudes of the target were revealed. Finally, we did not find evidence in any of the conducted studies that either personal or general BJW moderated the “beautiful is moral” stereotype.

Our study extends prior research on the “beautiful is moral” stereotype in several ways. First by demonstrating that the effect of attractiveness on moral character judgments is conditional and largely mediated by personal liking rather than attractiveness per se. Previous models, including the “halo effect”^1^ and moral beauty frameworks^7^ assume a direct and universal link between attractiveness and moral character. However, our findings challenge this assumption by showing that attractiveness primarily influences moral perception through attitudinal pathways rather than inherent biases. Earlier models may have overstated the direct connection between attractiveness and morality by overlooking the underlying factors and mechanisms that mediate this effect.

Second, we showed that the “beautiful is moral” stereotype was not moderated by general or personal belief in a just world in the US, Poland, and the UK. Therefore, contrary to the findings of Dion and Dion^11^, this result suggests that the “beautiful is moral” stereotype exists in people’s minds regardless of whether they believe the world is a just place. We also demonstrated that the “beautiful is moral” stereotype may exist in post-communist societies (e.g., Poland), which, in contrast to capitalist societies, are more suspicious, do not believe in a just world^55^, and present lower levels of system justification^56^. Thus, we may conclude that the “beautiful is moral” stereotype may exist in capitalist and post-communist societies, but so far, we have evidence that the stereotype is limited to the white faces of women.

Third, we showed that liking predict moral character judgments even when sociability and vanity are controlled, explain the extra variance, and is an independent mediator between attractiveness and moral character judgments. This supports the research of Han and Laurent^6^, proposing a third, probably the strongest mediator, together with sociability and vanity. We demonstrated that liking explain the impact of attractiveness on moral character judgments both alone and with sociability and vanity. Furthermore, we found that when accounting for liking, the effect of attractiveness on moral character judgments was eliminated, confirming strong ties between liking and morality.

Finally, Study 3 presented a surprising reversal of the “beautiful is moral” effect, where moderately attractive individuals were judged as having higher moral character than highly attractive individuals. This happened when we additionally manipulated liking toward the target. Confirming the attitudinal biasing power^17^, the similar target was judged as more moral than the dissimilar target. This reversal may be explained through the interplay of two factors.

First, previous studies^6^ suggest that highly attractive individuals may also be perceived as vain, which can counteract positive moral attributions. Our Study 3 findings align with this by showing that vanity ratings were significantly higher for highly attractive targets, suggesting that participants may have inferred a negative trait from extreme attractiveness. Second, the sample used in Study 3 was British, whereas Studies 1 and 2 used U.S. and Polish samples. Prior research (e.g^57^). , indicates that moral judgments are culturally influenced, and Western European contexts may prioritize humility over appearance-based moral attributions. Thus, our findings suggest that the “beautiful is moral” stereotype may be moderated by cultural variations in how attractiveness and humility are perceived as moral traits.

Further, reversal effects observed in Study 3 align with emerging research on stereotype reversals (e.g^15^) , which posits that individuals adjust stereotype application based on contextual cues. When attractiveness is perceived as excessive or linked to self-absorption (as indicated by higher vanity ratings), moral attributions may shift negatively. This complements work on the compensatory stereotype hypothesis, where individuals perceived as high in one domain (e.g., attractiveness) may be viewed as lower in another (e.g., morality) to maintain social balance^9^.

Eventually, another plausible explanation is that similarity manipulation reversed the “beautiful is moral” stereotype because of well-documented bias in person perception: assumed similarity effect^58^. When a person evaluates others, their self-assessment often influences how they perceive others. People assign higher moral worth to traits they believe they possess and judge those who share these traits more favorably. Conversely, they rate individuals lacking these traits more critically. Vonasch and Tookey^59^ demonstrate that self-serving biases shape moral assessments, leading individuals to perceive their own qualities as more virtuous while devaluing others’ differing attributes. Therefore, similarity manipulation might have impacted the perception of a moderately attractive face as more moral than a highly attractive face because participants rate their attractiveness as moderate, not high. In other words, participants assumed that a moderately attractive target was more similar to them than a highly attractive target when we made similarity salient. Future studies would do well investigating whether and how self-rated attractiveness moderates the “beautiful is moral” stereotype.

## Limitations, implications, and future directions

We recognize that our work may have some limitations and require further research. In two of the three studies, we did not find evidence that attractiveness impacts the perception of vanity, contradicting previous research by Han and Laurent^6^. However, we used some of the face images but not all of them from their study, and they also used descriptions of attractiveness as well as measures and manipulations of immoral behavior. Therefore, the discrepancy could result from the smaller number of stimuli used in our studies.

It is also important to stress that we used only White faces, which limits the generalizability of presented results. Indeed, white judges tend to react more negatively to faces that appear more stereotypically Black, associating them with negative traits^60^. Thus, future studies would do well by investigating if racial features impact the influence of attractiveness on morality, sociability, and vanity.

Another important discovery from our studies demonstrated that the “beautiful is moral” stereotype was limited to women targets. For men targets, attractiveness did not impact the perception of their morality. This finding is opposite to what was reported in previous studies (e.g^6^), while other studies used men and women targets but did not analyze gender effects (e.g^5^) . However, our findings align with existing research suggesting that physical attractiveness is more central to societal expectations of women than men, potentially leading to stronger associations between attractiveness and perceived morality for women targets^3^.

This gendered perception may stem from traditional gender roles that emphasize appearance for women, influencing moral character assessments. For example, research indicates that qualities such as physical attractiveness and nurturing behavior are highly valued in women. In contrast, traits like honesty, morality, and professional success are more emphasized for men^61^. Furthermore, studies have shown that men tend to dissociate sexual attraction from moral judgments more than women do, suggesting that women’s perceptions of morality are more influenced by attractiveness^62^. In summary, the gendered nature of the “beautiful is moral” stereotype may reflect deep-rooted societal norms that link women’s value more closely to their physical appearance, thereby influencing moral judgments based on attractiveness. Future research should explore the underlying mechanisms of this gender-specific effect.

We also did not find evidence that attractiveness impacts moral traits stronger than non-moral, as demonstrated by Klebl et al.^5^. This could be because we used different non-moral traits; however, as in Klebl and colleagues’^5^ research, we measured traits related to the dimension of warmth. In fact, if we look at the size of the effects of attractiveness on sociability (warmth) and moral character found in Studies 1 and 2, we can see that attractiveness had a stronger impact on sociability (*η²* = 0.14 and *η²* = 0.06, respectively) than on moral character judgments (*η²* = 0.03 and *η²* = 0.03, respectively). It seems important to establish in future studies when and how attractiveness impacts traits related to warmth and morality. Moreover, since we used the Face Research Lab London Set^35^ for attractiveness manipulation while Klebl et al.^6^ used the Chicago Face Database^63^, future studies could establish if cultural differences in attractiveness perception moderate moral character judgments.

Previous research has often treated BJW as a unidimensional construct, as in the study of Dion and Dion^11^, where BJW moderated the impact of attractiveness on goodness. However, Furnham^39^ highlighted the importance of distinguishing between personal and general just-world beliefs, noting that these dimensions can differentially predict attitudes and behaviors. In contrast to these suggestions, our studies did not support the hypothesis that personal or general belief in a just world moderates the “beautiful is moral” stereotype. One possibility of the absence of a moderating effect of BJW in our study may be attributed to the limitations of traditional BJW measures, which do not account for the multidimensionality of the construct. This aligns with recent critiques suggesting that conventional measures of BJW may lack specificity, thereby limiting their predictive power in specific contexts^38^. Integrating scales that distinguish different sources of justice, as Stroebe et al.^38^ suggested, may offer more significant understanding of the intricate relationship between BJW and societal perceptions. Thus, upcoming studies should explore using multidimensional scales that reflect various sources of justice to more precisely evaluate the impact of BJW on social perception.

## Conclusion

This study refines the “beautiful is moral” stereotype, showing that it applies primarily to women targets and does not necessarily imply vanity. The stereotype is stronger for warmth-related traits than for moral character. Notably, we observed a reversal effect when similarity information was introduced. Moderately attractive individuals were judged as more moral than highly attractive ones, suggesting that extreme attractiveness may sometimes reduce moral attributions. Additionally, perceived similarity led to higher moral judgments, while dissimilarity diminished them. These findings highlight the role of attitudes and social context in attractiveness-based moral perceptions. Future research should examine cultural differences, individual traits, and contextual moderators that shape when and how attractiveness influences moral judgments.

## Electronic supplementary material

Below is the link to the electronic supplementary material.

Supplementary Material 1

## Data Availability

The data are openly available at osf.io/c8d4g
