# Supplementary material for: Reevaluating the beautiful is moral stereotype by examining the impact of personal liking and belief in a just world
Source: Sci Rep. 2025 Apr 11;15:12387. doi: 10.1038/s41598-025-97022-2 (PMC11992105; doi:10.1038/s41598-025-97022-2)
Supplement: Supplementary file 1 — Supplementary Material 1 [file 41598_2025_97022_MOESM1_ESM.docx]

**Supplementary Materials, Additional Measures, and Analyses for:**

Reevaluating the “Beautiful is Moral” Stereotype by Examining the Impact of Personal Liking and Belief in a Just World

*Note.* Full verbatim instructions for each survey, stimuli used, R code and the .html output (statistical analyses results) are available on the Open Science Framework (<https://osf.io/c8d4g>).

**Additional Preregistered Analyses**

**Study 1**

For the exploratory analyses (preregistered), we ran two moderated mediation analyses following Hayes’ model 5, examining whether the two forms of BJW moderated the (direct) relation between attractiveness and moral character ratings, when mediated by liking (see full analysis output on OSF). The direct path in this model was not significantly moderated by either personal BJW, *b* = 0.05, SE = 0.04, R^2^ = 0.36, *F* (1, 781) = 2.01, *p* = .156, or general BJW, *b* = 0.03, SE = 0.04, R^2^ = 0.36, *F* (1, 782) = 0.69, *p* = .406.

**Study 2**

We ran the same moderated mediation analysis as in Study1. The direct path in this mediation model was not significantly moderated by personal BJW, *b* = 0.01, SE = 0.04, R^2^ = 0.44, *F* (1, 1515) = 0.03, *p* = .860, and was only marginally significant for general BJW, *b* = 0.07, SE = 0.04, R^2^ = 0.43, *F* (1, 1509) = 4.03, *p* = .045. Simple slopes analysis showed a significant positive effect of attractiveness on moral character at higher (+1 *SD*) levels of general BJW, *b* = 0.14, *SE* = 0.05, *p* = .007, 95% CI [0.04, 0.24], but not at average, *b* = 0.07, *SE* = 0.04, *p* = .080, 95% CI [-0.01, 0.14], or lower (-1 *SD*) levels of BJW, *b* = 0.01, *SE* = 0.05, *p* = .889, 95% CI [-0.11, 0.10]. .

**Study 3**

We hypothesized that liking would explain the impact of attractiveness on moral character above and beyond sociability and vanity. Table S1 shows that attitude was a significant positive predictor of moral character judgments, even after accounting for the effects of attractiveness, sociability, vanity and participant gender.

**Table S1**

*Linear Model Results with Predictors of Moral Character Judgments (Study 3)*

|  | Step 1 | | | | |  | Step 2 | | | | |
| --- | --- | --- | --- | --- | --- | --- | --- | --- | --- | --- | --- |
| Predictors | B | SE | 95% CI | Beta | *p* |  | B | SE | 95% CI | Beta | *p* |
| Attractiveness (high) | -0.08 | 0.05 | -0.17, 0.01 | -0.05 | .065 |  | -0.13 | 0.04 | -0.21, -0.05 | -0.08 | .002 |
| Sociability | 0.34 | 0.03 | 0.29, 0.39 | 0.35 | <.001 |  | 0.26 | 0.03 | 0.21, 0.31 | 0.27 | <.001 |
| Vanity | -0.26 | 0.02 | -0.30, -0.22 | -0.34 | <.001 |  | -0.20 | 0.02 | -0.24, -0.16 | -0.26 | <.001 |
| Participant gender (male) | -0.04 | 0.04 | -0.15, 0.02 | -0.04 | .146 |  | -0.02 | 0.04 | -0.10, 0.06 | -0.01 | .621 |
| Attitude (similar) |  |  |  |  |  |  | 0.14 | 0.04 | 0.05, 0.22 | 0.08 | .002 |
| Liking |  |  |  |  |  |  | 0.20 | 0.02 | 0.16, 0.24 | 0.28 | <.001 |
|  | N = 1087 | | | | |  | N = 1087 | | | | |
|  | *F*(4, 1082) = 110.15, *p* < .001 | | | | |  | *F*(6, 1080) = 109.11, *p* < .001 | | | | |
|  | R^2^ = 0.29/ R^2^ _adj_ = 0.29 | | | | |  | R^2^ = 0.38/ R^2^ _adj_ = 0.37 | | | | |

**Materials**

**Studies 1 and 2**

**Figure S1**

*Pictures of highly and moderately attractive men and women (Study 1 and Study 2)* *from Study 2a of Han and Laurent (2022).*


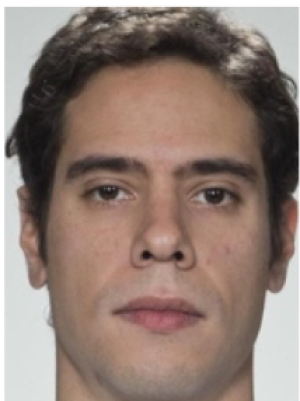


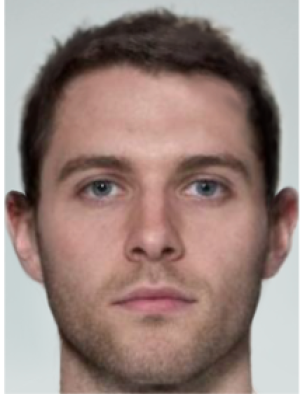


Highly attractive man

Moderately attractive man


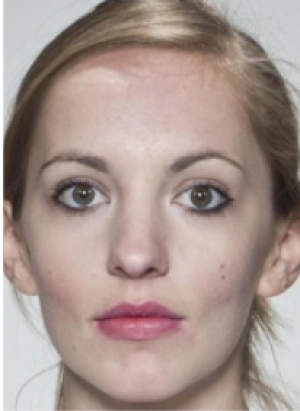

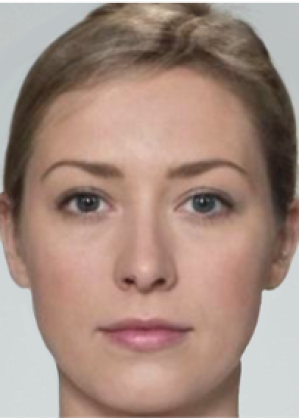


Moderately attractive woman

Highly attractive woman

**Study 3**

**Figure S2**

*Pictures of highly attractive and moderately attractive women (Study 3) from Study 2b of Han and Laurent (2022).*


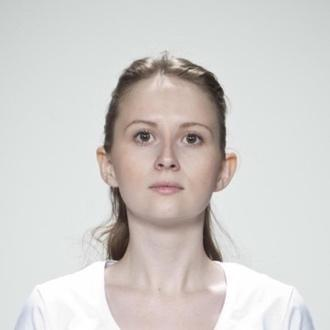

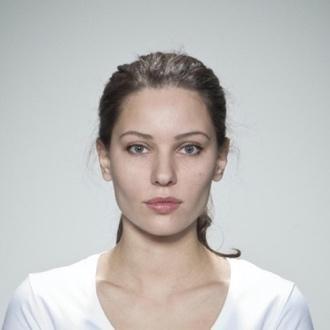


Highly attractive woman

Moderately attractive woman

**Similarity manipulation:**

List of preference questions (used as a similarity manipulation):

*“*Which do you prefer*? coffee/tea”,*

*“*Which do you prefer? *Reality show/sitcom”,*

*“*Which best describes you? *Dreamer/doer” ,*

*“*Which do you prefer?*Radio/Spotify”,*

*“*Which best describes you? *Spender/Saver”,*

*“*Which best describes you? *Morning glory/Night owl”,*

*“*What do you prefer? *Frozen yogurt/ice cream”,*

*“*Which do you prefer? *Big party/romantic dinner for 2”,*

*“*Do you? *Pay attention to details/pay attention to a big picture”,*

*“*Which do you prefer? *Mac/PC”,*

*“*Which one do you prefer to do? *Go with the flow/stick to the routine”,*

*“*Which best describes you? *Worry wart/worry free”,*

*“*Which do you prefer? *Museum of art/museum of natural history”,*

*“*Which do you prefer? *Shop alone/shop with others”,*

*“*Would you rather? *Watch TV/read a book”,*

*“*Which do you prefer? *Dark chocolate/milk chocolate”,*

*“*Which best describes you? *Sloppy/neat freak”.*

In the similar preferences condition, 14 answers from 17 mirrored participants’ responses. In the dissimilar preferences condition, 14 out of 17 answers were opposite to the participants’ responses.
